# Allergic lung inflammation alters neither susceptibility to *Streptococcus pneumoniae *infection nor inducibility of innate resistance in mice

**DOI:** 10.1186/1465-9921-10-70

**Published:** 2009-07-27

**Authors:** Cecilia G Clement, Michael J Tuvim, Christopher M Evans, Daniel M Tuvin, Burton F Dickey, Scott E Evans

**Affiliations:** 1 Department of Pulmonary Medicine, The University of Texas M. D. Anderson Cancer Center, 1515 Holcombe Boulevard, Houston, TX 77030, USA; 2 Center for Lung Inflammation and Infection, Institute of Biosciences and Technology, Texas A&M Health Science Center, 2121 Holcombe Boulevard, Houston, TX 77030, USA

## Abstract

**Background:**

Protective host responses to respiratory pathogens are typically characterized by inflammation. However, lung inflammation is not always protective and it may even become deleterious to the host. We have recently reported substantial protection against *Streptococcus pneumoniae *(pneumococcal) pneumonia by induction of a robust inflammatory innate immune response to an inhaled bacterial lysate. Conversely, the allergic inflammation associated with asthma has been proposed to promote susceptibility to pneumococcal disease. This study sought to determine whether preexisting allergic lung inflammation influences the progression of pneumococcal pneumonia or reduces the inducibilty of protective innate immunity against bacteria.

**Methods:**

To compare the effect of different inflammatory and secretory stimuli on defense against pneumonia, intraperitoneally ovalbumin-sensitized mice were challenged with inhaled pneumococci following exposure to various inhaled combinations of ovalbumin, ATP, and/or a bacterial lysate. Thus, allergic inflammation, mucin degranulation and/or stimulated innate resistance were induced prior to the infectious challenge. Pathogen killing was evaluated by assessing bacterial CFUs of lung homogenates immediately after infection, the inflammatory response to the different conditions was evaluated by measurement of cell counts of bronchoalveolar lavage fluid 18 hours after challenge, and mouse survival was assessed after seven days.

**Results:**

We found no differences in survival of mice with and without allergic inflammation, nor did the induction of mucin degranulation alter survival. As we have found previously, mice treated with the bacterial lysate demonstrated substantially increased survival at seven days, and this was not altered by the presence of allergic inflammation or mucin degranulation. Allergic inflammation was associated with predominantly eosinophilic infiltration, whereas the lysate-induced response was primarily neutrophilic. The presence of allergic inflammation did not significantly alter the neutrophilic response to the lysate, and did not affect the induced bacterial killing within the lungs.

**Conclusion:**

These results suggest that allergic airway inflammation neither promotes nor inhibits progression of pneumococcal lung infection in mice, nor does it influence the successful induction of stimulated innate resistance to bacteria.

## Introduction

Infectious pneumonia is the leading cause of premature death in the world [1-3], and *Streptococcus pneumoniae *is the primary cause of bacterial pneumonia [4]. The role of asthma in the development of pneumococcal pneumonia remains controversial, with possible bidirectional interactions between allergic airway inflammation and immune responses that are protective against bacteria. On one hand, there is an increasing literature showing that the innate immune system influences allergic airway inflammation [5], and bacterial colonization of neonates' airways is a risk factor for subsequent asthma [6]. On the other hand, asthma is a risk factor for invasive pneumococcal disease [7,8], and allergic airway inflammation has been found to diminish protective immunity to bacterial pneumonia [9]. Asthma might also contribute to pneumococcal susceptibility by other mechanisms. For example, asthma-associated changes in airway mucus may provide a sanctuary to pathogens [10], asthma treatments (e.g., corticosteroids) may impair native host defenses [11], or allergic inflammation may cause epithelial injury resulting in decreased production of defensive factors and/or impairment of barrier function [12].

We have recently reported that stimulation of lung innate immunity with an aerosolized lysate of non-typeable *Haemophilus influenzae *(NTHi) confers high-level protection against challenge with otherwise lethal inocula of *S. pneumoniae *[13]. Lysate-induced protection, called stimulated innate resistance (StIR), does not depend on recruited neutrophils, resident mast cells or alveolar macrophages, and is specific to the airway route of infection. The survival benefit correlates in magnitude and time with rapid pneumococcal killing within the lungs, and is associated with increased concentrations of numerous antimicrobial polypeptides in lung lining fluid. However, we have not previously tested whether StIR can be induced in the setting of acute allergic inflammation.

Given prior reports of asthma as a risk factor for invasive pneumococcal disease [7] and impaired host defenses of mice with allergic lung inflammation [9], we tested in mice whether preexisting allergic inflammation affected survival of pneumococcal pneumonia or the induction of StIR. We demonstrate here that induction of an asthmatic phenotype by transient allergic airway inflammation neither promotes nor inhibits progression of pneumococcal pneumonia in mice. Further, stimulation of mucin degranulation in allergically inflamed lungs does not affect progression of pneumococcal pneumonia, nor does allergic inflammation affect protection induced by pretreatment with inhaled NTHi lysate.

## Methods

### Animals

All experiments were performed using female, specific pathogen free, 5–8 week old BALB/c mice purchased from Harlan (Indianapolis, IN). Mice were handled in accordance with the policies of the Institutional Animal Care and Use Committee of the University of Texas-M. D. Anderson Cancer Center.

### Induction of allergic airway inflammation

Mice were sensitized to ovalbumin by four weekly intraperitoneal injections (20 μg ovalbumin Grade V, 2.25 mg alum in saline, pH 7.4; Sigma, St. Louis, MO), as described [14]. More than two weeks later, they were challenged for 30 min with an aerosol of 2.5% (wt/vol) ovalbumin in 0.9% saline supplemented with 0.02% (vol/vol) antifoam A silicon polymer (Sigma, St Louis, MO), using an AeroMist CA-209 nebulizer (CIS-US, Bedford, MA) driven by 10 l/min of 5% CO_2 _in room air to promote deep ventilation. To stimulate mucin secretion, ovalbumin sensitized mice were exposed to a 5 min ATP aerosol (100 mM) 3 days after ovalbumin aerosol challenge, as we have previously described to achieve maximal degranulation [14,15].

### Aerosolized bacterial lysate treatment

Frozen stock of non-typeable *Haemophilus influenzae *(NTHi) was grown on chocolate agar (Remel, Lenexa, KS), expanded in brain-heart infusion broth (Acumedia, Baltimore, MD) supplemented with 3.5 μg/ml NAD (Sigma), and disrupted with an EmulsiFlex C5 (Avestin, Mannheim, Germany), as described [13,16]. The protein concentration was adjusted to 2.5 mg/ml in saline by bicinchoninic assay (Pierce, Rockford, IL), and the lysate was frozen in 10 ml aliquots at -80°C. For treatment, a thawed aliquot was placed in an AeroMist CA-209 nebulizer (CIS-US) driven by 10 l/min 5% CO_2 _in air for 20 min. This resulted in aerosolization of 4 ml of lysate, with the protein concentration in residual lysate confirmed at 2.5 mg/ml. The nebulizer was connected by polyethylene tubing (30 cm × 22 mm) to a 10 liter polyethylene exposure chamber (approximately 12 × 7 × 8 inches), with an identical efflux tube with a low resistance microbial filter (BB50T, Pall, East Hills, NY) at its end vented to a biosafety hood.

### Pneumococcal pneumonia

As previously described [13], *S. pneumoniae *serotype 4 isolated from the blood of a patient with pneumonia was stored as frozen stock (1 × 10^9 ^CFU) in 20% glycerol in Todd-Hewett broth (Becton Dickinson, Franklin Lakes, NJ). Thawed stock was grown to logarithmic phase in Todd-Hewitt broth, then centrifuged at 4500 × *g *for 30 min at 4°C, washed and resuspended in PBS. Bacterial concentration was determined by plating serial dilutions onto blood-agar (Remel). For aerosolization, 10 ml of the bacterial suspension was nebulized in an identical exposure apparatus to that used for NTHi lysate treatment, 3 days after ovalbumin challenge, and immediately after ATP or NTHi lysate stimulation.

### Lung bacteria quantification

Immediately upon completion of the nebulization of the *S. pneumoniae*, infected mouse lungs were extracted following induction of deep anesthesia, homogenized in 1 ml of PBS using a 2 ml tissue grinder (Kontes, Vineland, NJ), then serially diluted onto blood-agar plates with tryptic soy agar (Remel), and incubated overnight at 37°C in 5% CO_2_.

### Bronchoalveolar lavage fluid

Bronchoalveolar lavage (BAL) fluid was obtained by instilling and collecting two aliquots of 1 ml each of PBS through a luer stub adapter cannula (Becton Dickinson) inserted through rings of the exposed trachea of euthanized mice 18 h after challenge with ovalbumin, NTHi lysate, ATP, and *S. pneumoniae*. Total leukocyte count was determined with a hemacytometer (Hauser Scientific, Horsham, PA), and differential count by cytocentrifugation of 300 μl of BAL fluid at 2,000 rpm for 5 min, followed by Wright-Giemsa staining.

### Statistical methods

Proportions of mice surviving *S. pneumoniae *challenge were compared using Fisher's exact test at 7 days after infection. Student's t test was used to examine the differences between the mean bacterial counts in lung tissue for the various conditions, as well as for comparison of leukocyte counts in BAL fluid analysis.

## Results

### Allergic lung inflammation neither promotes nor suppresses mortality from pneumococcal pneumonia in mice

**Figure 1:**
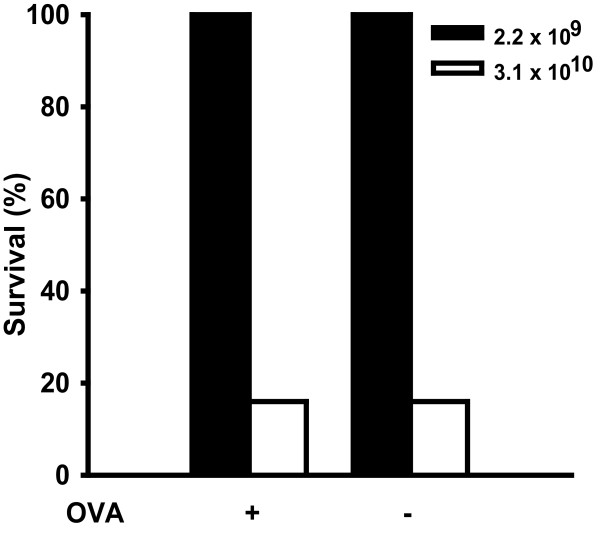
**Allergic lung inflammation neither promotes nor suppresses *S. pneumoniae *pneumonia-associated mortality**. Survival of mice seven days after challenge with high or low dose *S. pneumoniae *in the presence or absence of allergic inflammation (6 mice/group).

Survival was assessed 7 days after aerosol challenge with *S. pneumoniae *in the presence or absence of allergic inflammation (Fig. 1). A high dose (3.1 × 10^10 ^CFU/ml) of *S. pneumoniae *was used to uncover a protective effect of allergic inflammation, and a low dose (2.2 × 10^9 ^CFU/ml) was used to uncover increased susceptibility. Ovalbumin sensitization and challenge neither promoted nor suppressed survival of mice challenged with either dose of *S. pneumoniae*.

### Allergic inflammation and induced mucin secretion affect neither survival nor whole lung bacterial counts in pneumococcal pneumonia

**Figure 2:**
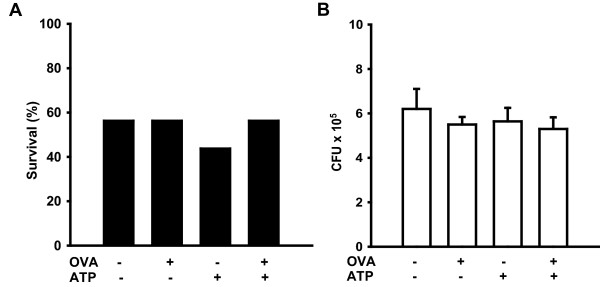
**Allergic inflammation and induced secretion affect neither survival nor pathogen burden in *S. pneumoniae *pneumonia**. **(A) **Survival of ovalbumin-sensitized mice 7 days after challenge with intermediate dose (1.6 × 10^10 ^CFU/ml) *S. pneumoniae *after induction of allergic inflammation by inhaled ovalbumin and/or secretion by inhaled ATP, as indicated (16 mice/group). There was no statistically significant difference in survival in any group compared to naive (OVA-, ATP-) mice. **(B) **Bacterial counts of lung homgenates immediately after pneumococcal challenge (1.6 × 10^10 ^CFU/ml) of ovalbumin-sensitized mice, with or without induction of allergic inflammation and/or secretion (3 mice/group).

In the setting of allergic inflammation with airway epithelial mucous metaplasia, ATP exposure induces mucin degranulation ("mucus hypersecretion") [14]. Impacted airway lumenal mucus can protect bacteria from host defenses and induce biofilm formation, so we tested whether mucus hypersection altered the progression of pneumococcal pneumonia. ATP can also induce exocytosis in airway secretory cells without mucous metaplasia and in alveolar type II cells [17], as well as induce inflammation directly or through breakdown products such as adenosine [18,19] so, we tested whether ATP alone had any effects. Neither allergic inflammation, ATP-induced secretion, nor the combination had any effect on survival of pneumococcal pneumonia (Fig. 2A). We also tested whether 100 mM aerosolized ATP induced airway inflammation, and found no increase in BAL leukocytes (data not shown).

Survival of pneumococcal challenge is tightly correlated with the lung pathogen burden [13]. Consistent with our survival data, exposure of ovalbumin-sensitized mice to inhaled ovalbumin, ATP or both resulted in no significant changes in whole lung bacterial CFUs immediately after infection with *S. pneumoniae *(Figure 2B).

### NTHi lysate protects against pneumococcal pneumonia and induces pathogen killing despite the presence of allergic inflammation

**Figure 3:**
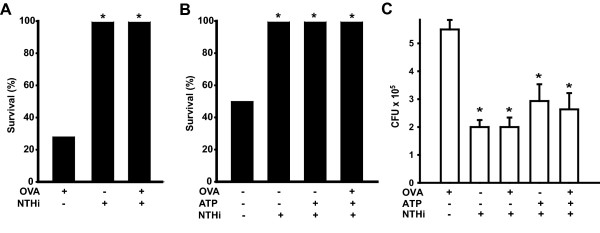
**NTHi lysate protects against *S. pneumoniae *pneumonia in the presence of non-protective allergic inflammation**. **(A) **Survival of ovalbumin-sensitized mice seven days after challenge with *S. pneumoniae *(6.8 × 10^10 ^CFU/ml) following inhalation of ovalbumin and/or NTHi lysate prior to challenge (7 mice/group, *p = 0.01). **(B) **Survival of ovalbumin-sensitized mice seven days after challenge with *S. pneumoniae *(2.0 × 10^10 ^CFU/ml) following inhalation of ovalbumin and/or NTHi lysate, with or without inhaled ATP exposure prior to challenge (6 mice/group, *p < 0.05). **(C) **Bacterial counts of lung homogenates immediately after pneumococcal challenge (1.2 × 10^10 ^CFU/ml) of ovalbumin-sensitized mice, with or without induction of allergic inflammation and/or secretion and protective lysate exposure (3 mice/group, *p < 0.001 compared to mice not receiving NTHi treatment, mean ± SEM). OVA: inhaled ovalbumin 3 d prior to infection; ATP: inhaled ATP 5 min prior to infection; NTHi: inhaled NTHi lysate 1 d prior to infection. Figures shown are representative of at least three experiments.

In addition to constitutive innate immune defenses of the lungs, innate defenses can be powerfully induced [13,20,21]. We sought to determine whether bacteria-protective innate immunity could be induced in the setting of preexisting allergic inflammation by testing whether resistance to pneumococcus could be stimulated by aerosolized NTHi lysate following ovalbumin sensitization and challenge (Fig. 3A). Because stimulated resistance could potentially be abrogated by exhaustive exocytosis and clearance of important defense mediators, or by secreted mucins sequestering defense mediators or providing a sanctuary for pathogens, we also tested the effect of ATP exposure (Fig. 3B). As we have previously shown [13], a single NTHi lysate pretreatment conferred complete protection against pneumococcal challenge. This protection was not attenuated by the induction of allergic inflammation, induction of acute secretion with ATP, or both.

We have previously found that StIR correlates closely with rapid bacterial killing within the lungs, and that reduced pathogen levels in the lungs immediately after infection correlate with decreased pathogen levels in the lungs, blood and spleen at later time points [13,20,21]. While pretreatment with NTHi lysate was associated with significant reductions in the number of bacterial CFU cultured from whole lung homogenates immediately after the infectious challenge, we did not detect a statistically significant effect of the allergic inflammation/secretion status (OVA, ATP or both) on the inducibility of rapid bacterial killing (Fig. 3C).

### NTHi lysate treatment induces significant airway neutrophilia in the presence or absence of allergic inflammation

**Figure 4:**
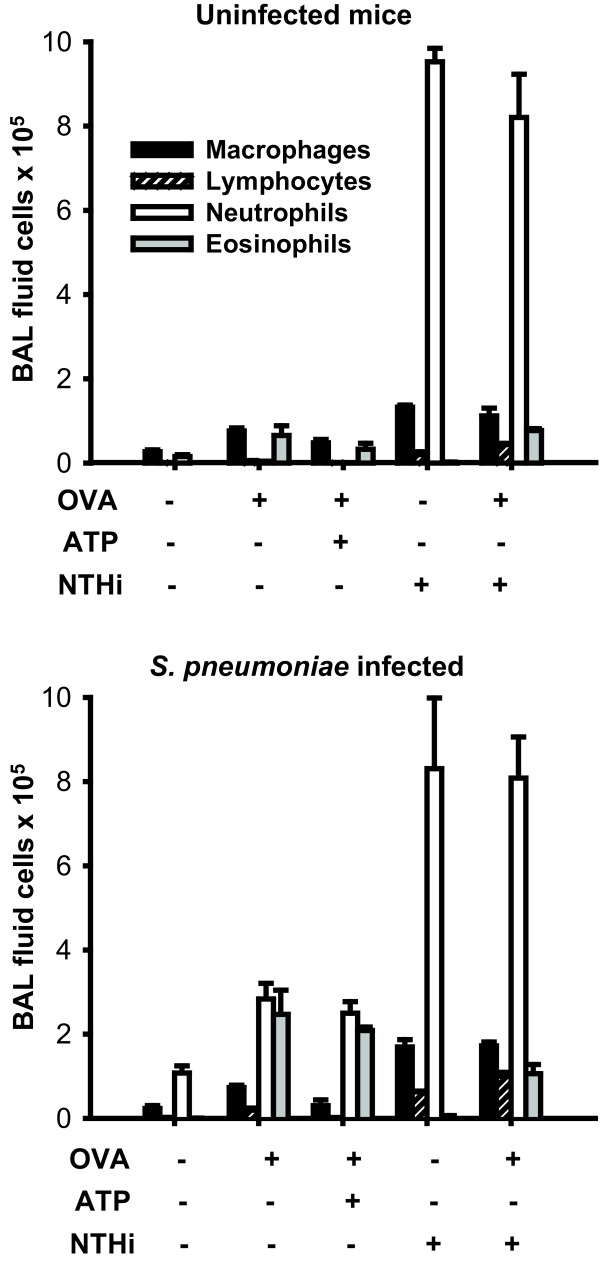
**Leukocyte infiltration following various inflammatory stimuli**. Differential cell counts were performed on bronchoalveolar lavage fluid from mice following treatment with different combinations of inflammatory stimuli without (upper panel) or with (lower panel) infection with *S. pneumoniae *(3.2 × 10^10 ^CFU/ml) 18 h earlier. The experiments were performed simultaneously with identical treatments, except for the infectious challenge. (3 mice/group, mean ± SEM).

In order to better characterize the lungs' responses to various inflammatory stimuli, we characterized the cellular influx of the airway lining fluid collected by BAL. As shown in Figure 4, the allergic and bacteria-protective stimuli induced influx of distinctly different cell populations. Exposure of sensitized mice to inhaled ovalbumin induced modest increases in macrophages and eosinophils, whereas treatment with NTHi lysate primarily induced infiltration with neutrophils. These patterns persisted when both treatments were applied to the same animal. For example, eosinophils increased following OVA challenge (0 detected eosinophils at baseline vs. 0.65 × 10^5 ^eosinophils after challenge, p = 0.047), whereas there was no difference detected when OVA challenged mice were compared to those challenged with OVA then treated with NTHi lysate (0.77 × 10^5 ^eosinophils, p = 0.64). Similarly, the brisk induction of neutrophils after NTHi lysate treatment (0.15 × 10^5 ^PMNs at baseline vs. 9.52 × 10^5 ^PMNs after treatment, p < 0.0001) was not significantly altered by the presence of allergic inflammation (8.20 × 10^5 ^PMNs, p = 0.29).

We have previously shown that, while lysate-induced resistance does not rely on neutrophils for protection, NTHi lysate treatment induces a much more robust lung neutrophilia that does *S. pneumoniae *[13]. We again found that infection with *S. pneumoniae *did not alter the entry of neutrophils into airspaces of the lungs following NTHi lysate treatment (9.52 × 10^5 ^PMNs after lysate alone vs. 8.27 × 10^5 ^PMNswhen *S. pneumoniae *infection followed lysate treatment, p = 0.51). Furthermore, NTHi lysate induced neutrophilia in the setting of infection was not significantly altered by allergic inflammation (8.07 × 10^5 ^PMNs, p = 0.91). We did, however, identify a statistically significant further increase in eosinophils when OVA challenged mice were infected with *S. pneumoniae *(0.65 × 10^5 ^eosinophils vs. 2.50 × 10^5 ^eosinophils, p = 0.04), perhaps reflecting the antibacterial function of eosinophils.

## Discussion

Asthma is a chronic disease of the lungs, associated with recurrent allergic inflammation and at least partially reversible airflow limitation [22]. According to the Centers for Disease Control and Prevention, the incidence of asthma in the United States has steadily increased to nearly 8% of the population in the past two decades . The reason for this trend is an issue of ongoing debate, but the finding of asthma as a risk factor for serious pneumococcal disease [7,8] brings great relevance to the understanding of how allergic airways inflammation affects the response to infection. Despite previous reports of impaired host responses to bacteria in the setting of allergic inflammation [9], we have shown here that transient induction of allergic inflammation neither promotes lethality in an experimental model of pneumococcal pneumonia, nor precludes the induction of pneumococcus-protective StIR.

Inflammation generated in the lungs can be induced by diverse stimuli, yet, as we have shown here, the nature of the stimulus markedly affects the character of the inflammatory response and whether it is beneficial to survival of microbial challenge. Our data indicate that only a subset of inflammatory responses are protective against pneumococcal pneumonia, and allergic inflammation is not part of this subset. Whether allergic inflammation is protective in other settings (e.g., helminth infections) remains to be seen [23,24]. The ovalbumin experimental model recapitulates many features of clinical asthma, including inflammatory cell infiltration, mucin overproduction, and airflow obstruction [25], yet we demonstrate no effect on survival of pneumococcal pneumonia. These data indicate that neither the polarization of the inflammatory response towards eosinophilic Type 2 immunity nor the marked overproduction and acute secretion of mucin is associated with worsened survival of pneumococcal infection of the lungs.

Beisswenger and colleagues previously reported that induction of Type 2 inflammation resulted in diminished anti-pseudomonal defenses [9]. They found that applying IL-4 and/or IL-13 to bronchial epithelial cells or inducing allergic inflammation in BALB/c mice resulted in higher post-challenge pathogen burdens. We found no such effect on lung pneumococcal CFUs immediately after infection in the presence of allergic inflammation, despite findings comparable to theirs in terms of the ovalbumin- and infection-induced lung eosinophilia. There are several differences between their model and ours, including the interval between sensitization and challenge, the means of maintaining a control group (we sensitize all mice and aerosolize ovalbumin or PBS, whereas they inject ovalbumin or PBS prior to aerosolizing ovalbumin to all mice), and the interval between challenge and pathogen burden assessment. The different pathogens used in the two studies is notable insofar as they are recognized by different complements of pattern recognition receptors, and allergic inflammation may exert a more profound effect on host responses to the Gram-negative pathogen [26]. However, the relevant difference between our studies may relate to the size of the inoculum. Beisswenger and colleagues report an increase in pathogen burden from around 75 CFU/mg lung to around 125 CFU/mg lung with induction of allergic inflammation. We found no pneumococcal CFU difference in the presence or absence of allergic inflammation, describing approximately 6 × 10^5 ^CFU/ml lung homogenate. It is possible that they identified a statistically significant difference at these very low pathogen concentrations that has limited bearing on progression to severe lung disease or death. In our experience, even the mice with 125 CFU/mg lung would be expected to remain healthy and fully clear the infection. Concordant with the reports of Beisswenger and colleagues, we observed in our previous studies [13,20,21] a critical role for the respiratory epithelium in innate protection of the lungs, associated with elaboration of numerous antimicrobial peptides. Yet, here we did not observe any influence of allergic inflammation on the ability of epithelial cells to protect against pneumococcal pneumonia *in vivo*.

More recently, Kang and colleagues reported that OVA sensitized and challenged BALB/c mice were less likely to develop pneumococcal pneumonia (as defined by bioluminescent assay) than were mice without allergic inflammation [27]. Interestingly, while they did not find intergroup differences in T_H_2 cytokines 7 days after OVA challenge, elevated IL-4 levels were associated with increased risk of pneumonia, independent of sensitization status. Our results are difficult to directly compare to those of Kang due to differences in the allergic model, the route and size of pneumococcal inoculum, and the means and timing of the outcome assessment. However, it is conceivable that differences in pulmonary deposition of bioluminescent pathogens relate more to upper airway inflammation prior to intranasal infection than to changes in the lower respiratory tract. That said, Yousefi and colleagues have recently characterized antibacterial eosinophil responses [28], so Kang may have detected a modestly protective eosinophilic effect that is dissociated from T_H_2 cytokine elaboration.

We have previously shown that exposure of mice to inhaled NTHi lysate stimulates innate resistance to bacterial pneumonia, a phenomenon associated with striking inflammation [13,16,20,21]. In this work, we demonstrate that the concurrent presence of allergic inflammation does not significantly influence the neutrophilic influx induced by inhalational exposure to NTHi lysate. More importantly, the preexistence of allergic inflammation did not abrogate the improved host survival associated with NTHi lysate. We also found that while NTHi lysate exposure promoted a rapid reduction in lung CFUs immediately after infection, treatment with inhaled ovalbumin and/or ATP had no such effect. Furthermore, concomitant allergic inflammation did not preclude the inducibility of rapid bacterial killing by lysate treatment. By limiting the initial bacterial burden in the lung, StIR appears to prevent the septicemia and death associated with uncontrolled lung infections. Though we have observed pathogen killing by multiple lung epithelial cell types, it is not clear whether treatment-related alterations in pathogen deposition within the lungs contributes to the protective effect. We have previously shown that innate resistance can be induced in the absence of the leukocytes commonly associated with bacterial protection [13]. Here we show that it can also be induced in the presence of preexisting inflammation that is not protective against bacteria. Together, these findings suggest the inducibility of innate defenses in a broad range of clinical scenarios.

Our experimental observations do not provide an explanation of the findings of Talbot and colleagues that a diagnosis of asthma is an independent risk factor for serious pneumococcal disease [7,8]. Potential reasons for this include the possibility that the animal model does not adequately approximate clinical asthma, that the primary endpoints were different (bloodstream infection in patients versus death in mice), or that the particular features of asthma we tested are not the cause of the risk identified by Talbot and colleagues. We focused on deviation of immune responses, stimulated innate resistance, and acute mucus hypersecretion. Other possibilities include disrupted epithelial barrier function in chronic clinical asthma, suppression of immune responsiveness by treatment of asthma with corticosteroids or other drugs, creation of a sanctuary for bacterial growth in airways with chronically impacted mucus, or the existence of a common cause for both asthma and pneumococcal susceptibility. Regarding the last possibility, while all patients in the study of Talbot and colleagues were of a socioeconomic status that qualified for government-supported health care coverage, the pneumococcal cases had many differences from the control subjects besides the asthma rate, including ethnicity and medical co-morbidities. In statistically correcting for those differences, it is possible that an additional association was masked. Alternatively, a common genetic cause of both asthma and pneumococcal susceptibility could be present in patients but not tested in our model.

## Conclusion

We have tested in mice several possible mechanistic causes of the epidemiologic finding that asthma is a risk factor for invasive pneumococcal disease. We find that the immune deviation of allergic lung inflammation does not suppress baseline or induced antibacterial innate defenses, nor does acute mucin hypersecretion. Further, the finding that allergic inflammation does not preclude the induction of protective innate resistance expands the clinical scenarios in which exploitation of this phenomenon might be therapeutically beneficial.

## Competing interests

CGC, MJT and BFD are the inventors of the subject matter disclosed in the patent application "Compositions and Methods for Stimulation of Lung Innate Immunity" filed by the Board of Regents of the University of Texas System. MJT, BFD and SEE own equity in Pulmotect, Inc., that is developing strategies to induce innate resistance to protect against pneumonia. CME and DMT have no competing interests to declare.

## Authors' contributions

CGC participated in development and performance of the NTHi treatment and infectious experiments. MJT participated in development and performance of the NTHi treatment and infectious experiments. CME participated in the development and performance of the allergic inflammation experiments. DMT participated in development and performance of the NTHi treatment and infectious experiments. BFD participated in design of experiments and drafting of the manuscript. SEE participated in data analysis and drafting of the manuscript.

## References

[B1] (2004). The World Health Report 2004 – Changing History.

[B2] File TM (2003). Community-acquired pneumonia. Lancet.

[B3] Joos L, Tamm M (2005). Breakdown of pulmonary host defense in the immunocompromised host: cancer chemotherapy. Proc Am Thorac Soc.

[B4] Advisory Committee on Immunization Practices (2000). Preventing Pneumococcal Disease Among Infants and Young Children. Morbid Mortal Week Rev.

[B5] Hammad H, Lambrecht BN (2008). Dendritic cells and epithelial cells: linking innate and adaptive immunity in asthma. Nat Rev Immunol.

[B6] Bisgaard H, Hermansen MN, Buchvald F, Loland L, Halkjaer LB, Bonnelykke K, Brasholt M, Heltberg A, Vissing NH, Thorsen SV (2007). Childhood asthma after bacterial colonization of the airway in neonates. N Engl J Med.

[B7] Talbot TR, Hartert TV, Mitchel E, Halasa NB, Arbogast PG, Poehling KA, Schaffner W, Craig AS, Griffin MR (2005). Asthma as a risk factor for invasive pneumococcal disease. N Engl J Med.

[B8] Juhn YJ, Kita H, Yawn BP, Boyce TG, Yoo KH, McGree ME, Weaver AL, Wollan P, Jacobson RM (2008). Increased risk of serious pneumococcal disease in patients with asthma. J Allergy Clin Immunol.

[B9] Beisswenger C, Kandler K, Hess C, Garn H, Felgentreff K, Wegmann M, Renz H, Vogelmeier C, Bals R (2006). Allergic airway inflammation inhibits pulmonary antibacterial host defense. J Immunol.

[B10] Tunney MM, Field TR, Moriarty TF, Patrick S, Doering G, Muhlebach MS, Wolfgang MC, Boucher R, Gilpin DF, McDowell A (2008). Detection of anaerobic bacteria in high numbers in sputum from patients with cystic fibrosis. Am J Respir Crit Care Med.

[B11] Calverley PM, Anderson JA, Celli B, Ferguson GT, Jenkins C, Jones PW, Yates JC, Vestbo J (2007). Salmeterol and fluticasone propionate and survival in chronic obstructive pulmonary disease. N Engl J Med.

[B12] Holgate ST (2008). Pathogenesis of asthma. Clin Exp Allergy.

[B13] Clement CG, Evans SE, Evans CM, Hawke D, Kobayashi R, Reynolds PR, Moghaddam SJ, Scott BL, Melicoff E, Adachi R (2008). Stimulation of lung innate immunity protects against lethal pneumococcal pneumonia in mice. Am J Respir Crit Care Med.

[B14] Evans CM, Williams OW, Tuvim MJ, Nigam R, Mixides GP, Blackburn MR, DeMayo FJ, Burns AR, Smith C, Reynolds SD (2004). Mucin is produced by clara cells in the proximal airways of antigen-challenged mice. Am J Respir Cell Mol Biol.

[B15] Tuvim MJ, Mospan AR, Burns KA, Chua M, Mohler PJ, Melicoff E, Adachi R, Ammar-Aouchiche Z, Davis CW, Dickey BF (2009). Synaptotagmin 2 couples mucin granule exocytosis to Ca2+ signaling from endoplasmic reticulum. J Biol Chem.

[B16] Moghaddam SJ, Clement CG, De la Garza MM, Zou X, Travis EL, Young HW, Evans CM, Tuvim MJ, Dickey BF (2008). Haemophilus influenzae lysate induces aspects of the chronic obstructive pulmonary disease phenotype. Am J Respir Cell Mol Biol.

[B17] Rice WR, Singleton FM (1986). P2-purinoceptors regulate surfactant secretion from rat isolated alveolar type II cells. Br J Pharmacol.

[B18] Idzko M, Hammad H, van Nimwegen M, Kool M, Willart MA, Muskens F, Hoogsteden HC, Luttmann W, Ferrari D, Di Virgilio F (2007). Extracellular ATP triggers and maintains asthmatic airway inflammation by activating dendritic cells. Nat Med.

[B19] Mohsenin A, Blackburn MR (2006). Adenosine signaling in asthma and chronic obstructive pulmonary disease. Curr Opin Pulm Med.

[B20] Evans SE, Scott BL, Clement CG, Pawlik J, Bowden MG, Hook M, Kontoyiannis DP, Lewis RE, LaSala PR, Peterson JW (2009). Stimulation of lung innate immunity protects mice broadly against bacterial and fungal pneumonia. Am J Respir Cell Molec Biol.

[B21] Tuvim MJ, Evans SE, Clement CG, Dickey BF, Gilbert BE (2009). Augmented lung inflammation protects against influenza A pneumonia. PLoS ONE.

[B22] Holt PG, Macaubas C, Stumbles PA, Sly PD (1999). The role of allergy in the development of asthma. Nature.

[B23] Reese TA, Liang HE, Tager AM, Luster AD, Van Rooijen N, Voehringer D, Locksley RM (2007). Chitin induces accumulation in tissue of innate immune cells associated with allergy. Nature.

[B24] Anthony RM, Rutitzky LI, Urban JF, Stadecker MJ, Gause WC (2007). Protective immune mechanisms in helminth infection. Nat Rev Immunol.

[B25] Tomkinson A, Cieslewicz G, Duez C, Larson KA, Lee JJ, Gelfand EW (2001). Temporal association between airway hyperresponsiveness and airway eosinophilia in ovalbumin-sensitized mice. Am J Respir Crit Care Med.

[B26] Ueno K, Koga T, Kato K, Golenbock DT, Gendler SJ, Kai H, Kim KC (2008). MUC1 mucin is a negative regulator of toll-like receptor signaling. Am J Respir Cell Mol Biol.

[B27] Kang CI, Rouse MS, Patel R, Kita H, Juhn YJ (2009). Allergic airway inflammation and susceptibility to pneumococcal pneumonia in a murine model with real-time in vivo evaluation. Clin Exp Immunol.

[B28] Yousefi S, Gold JA, Andina N, Lee JJ, Kelly AM, Kozlowski E, Schmid I, Straumann A, Reichenbach J, Gleich GJ (2008). Catapult-like release of mitochondrial DNA by eosinophils contributes to antibacterial defense. Nat Med.

